# Tinnitus and Self-Perceived Hearing Handicap in Firefighters: A Cross-Sectional Study

**DOI:** 10.3390/ijerph16203958

**Published:** 2019-10-17

**Authors:** Samson Jamesdaniel, Kareem G. Elhage, Rita Rosati, Samiran Ghosh, Bengt Arnetz, James Blessman

**Affiliations:** 1 Institute of Environmental Health Sciences, Wayne State University, Detroit, MI 48202, USA; kareem.elhage@wayne.edu (K.G.E.); ad6628@wayne.edu (R.R.); 2 Department of Family Medicine and Public Health Sciences, Wayne State University, Detroit, MI 48201, USA; sghos@med.wayne.edu (S.G.); james.blessman@wayne.edu (J.B.); 3 Department of Family Medicine, College of Human Medicine, Michigan State University, Grand Rapids, MI 49503, USA; arnetzbe@msu.edu

**Keywords:** tinnitus, hearing loss, ototoxicity, firefighters, environmental exposures, noise, lead

## Abstract

Firefighters are susceptible to auditory dysfunction due to long-term exposure to noise from sirens, air horns, equipment, and tools used in forcible entry, ventilation, and extrication. In addition, they are exposed to ototoxic chemicals, particularly, during overhaul operations. Studies indicate that 40% of firefighters have hearing loss in the noise-sensitive frequencies of 4 and 6 kHz. Noise-induced hearing loss (NIHL) is often accompanied by tinnitus, which is characterized by ringing noise in the ears. The presence of phantom sounds can adversely affect the performance of firefighters. However, there has been limited research conducted on the prevalence of tinnitus in firefighters. We enrolled firefighters from Michigan, with at least 5 years of continuous service. The hearing handicap inventory for adults (HHIA) was used to determine the difficulty in hearing perceived by the firefighters and the tinnitus functional index (TFI) was used to determine the severity of tinnitus. Self-perceived hearing handicap was reported by 36% of the participants, while tinnitus was reported by 48% of the participants. The TFI survey indicated that 31% perceived tinnitus as a problem. More importantly, self-perceived hearing handicap was significantly associated with the incidence of tinnitus in firefighters, suggesting a potential link between occupational exposure to ototraumatic agents and tinnitus in firefighters.

## 1. Introduction

Hearing loss, the third-most common chronic physical condition in American adults, can significantly affect quality of life and productivity [1]. Approximately 15% of Americans between the ages of 20 and 69 have high frequency hearing loss (National Institutes of Health/National Institute on Deafness and Other Communication Disorders) and about 12% of the U.S. working population has hearing difficulty (National Institute for Occupational Safety and Health). Exposure to ototraumatic agents, such as noise, chemicals, and heavy metals, in environmental and occupational settings, is a critical factor in acquired hearing loss. Moreover, the synergistic interaction between some of these ototraumatic agents can potentiate auditory dysfunction in susceptible individuals. First responders are extremely vulnerable to such adverse synergistic effects, as they are frequently exposed to various combinations of environmental risk factors in their day-to-day activities. In particular, firefighters are at risk of hearing impairment due to long-term exposure to occupational noise from sirens, water pumps, saws, and other equipment that make excessive noise [2]. They are also exposed to ototoxicant chemicals and metals, such as benzene, carbon monoxide, formaldehyde, lead, cadmium, mercury, and arsenic, that emanate from burning buildings [3]. In addition to hearing loss, occupational exposure to such ototraumatic agents can also lead to tinnitus, which is usually characterized by a ringing noise in the ears, in the absence of any external sound.

Tinnitus has been reported to often accompany sensory neural hearing loss induced by noise exposure, as well as aging. In the absence of normal auditory signals from the cochlea, the central auditory system undergoes abnormal neuroplastic changes, which may downregulate centrally-mediated inhibition and cause tinnitus [4]. Enhanced neuronal firing in the central auditory pathway has been detected in tinnitus. The incidence of tinnitus in the adult population of the United States is 10%–15%, as approximately 50 million US adults reported having tinnitus [5]. However, the prevalence rate is likely to be much higher in vulnerable populations, such as first responders, as they are often exposed to many of the risk factors. Although a recent study indicated that 52% of firefighters in San Francisco reported some degree of tinnitus [6], it is not clear whether the prevalence of tinnitus is at such a high levels among all firefighters. Nevertheless, depending on the frequency and amplitude, the presence of a phantom sound can adversely affect the performance of firefighters, as it can mask some of the auditory cues required to take appropriate actions in life-threatening situations. Particularly, in situations where heavy smoke impairs vision, they must rely on their hearing to make critical decisions. As tinnitus can be highly bothersome and cause anxiety, depression, stress, and sleep disturbance, it can compromise their efficiency. Despite these significant implications, very few studies have investigated the incidence of tinnitus in firefighters.

Urban areas, such as Detroit, were heavily industrialized in the past and have an older housing stock. A recent study indicated that the levels of lead and cadmium are at much higher concentrations in southeast Michigan, probably due to leaking and spillage of chemicals from various industries [7]. Consequently, firefighters in such areas are one among the extremely vulnerable population as they are exposed to multiple environmental insults that can cause hearing impairment and tinnitus. A thorough understanding of the incidence of tinnitus in these firefighters would eventually lead to the design of effective remediation measures, which in turn will minimize potential work-related auditory dysfunction and injuries. Therefore, we employed a cross-sectional study to determine the presence and severity of tinnitus and self-perceived hearing handicap in firefighters from a large municipal fire department in Michigan. A cohort of 42 firefighters was recruited and the presence of tinnitus was analyzed using the tinnitus functional index (TFI) questionnaire while hearing handicap was assessed using the hearing handicap inventory for adults (HHIA) questionnaire.

## 2. Materials and Methods 

### 2.1. Study Design

A cross-sectional design was used for this exploratory study, which was approved by the Wayne State University’s Institutional Research Board (IRB # 064814MP2E & 084017MP2F). Firefighters were recruited from several engine houses in Michigan. Invitations to participate in this study were given through the Fire Department, as well as through the Fire Fighters Association. Research personnel also visited engine houses to introduce the study. Firefighters expressed their interest in participating in the study via telephone, email, or in-person during engine house visits. Three different survey forms were distributed to the recruited participants to determine prior exposure to ototraumatic agents, self-perceived hearing handicap, and incidence of tinnitus. The questionnaires were completed by firefighters at the engine houses or in the clinic, in the presence of research personnel, so that any clarification needed for answering the questions was provided immediately. Career firefighters, who have worked for a minimum of 5 continuous years, were included in the study, while those with a familial history of hearing loss, middle ear disease, other serious ailments, or large differences in the hearing threshold (>40 dB) between left and right ear were excluded from the study. Pure-tone audiometry was used to identify large differences in hearing thresholds. Written consent was obtained prior to participation in the study.

### 2.2. Prior Exposures Survey

Potential exposure to ototraumatic agents and environmental factors that influence hearing was assessed using a “History of Prior Exposures” questionnaire. In addition to demographic information, this questionnaire was used to obtain information regarding the number of years in service as a firefighter, job title, exposure to non–work noise (loud music, auto repair, wood working/cutting, metal working, snowmobiling, motor-biking, boating, hunting/target practicing, auto racing, noisy equipment, other exposures), use of medications, serious illness (mumps, meningitis, measles), injuries to ear or head, family history of hearing problems, use of implants or hearing aids, other ear problems (drainage, infection, aches, ringing), exposure to solvents or vibration, and habits (smoking, alcohol, caffeine, drugs). This information was used to identify firefighters who have been exposed to multiple risk factors so that they could be excluded from the analysis.

### 2.3. Hearing Handicap Inventory for Adults (HHIA) Survey

Self-perceived hearing handicap in firefighters was assessed using the HHIA survey [8]. This is a 25-item self-assessment scale, composed of a 13-item emotional subcategory and a 12-item social/situational subcategory. Users were asked to answer “yes” (4 points), “sometimes” (2 points) or “no” (no point) for each question according to what they thought to be more appropriate to their condition. The total score ranges from 0 to 100. This survey has been demonstrated to have an excellent test-retest reliability (r = 0.97) and high internal consistency reliability with a Cronbach’s alpha value of 0.93 [9]. The HHIA survey has been used previously to assess hearing handicap in firefighters [6]. Therefore, we employed the same survey to enable the comparison of our findings with these studies.

### 2.4. Tinnitus Functional Index (TFI) Survey

The TFI questionnaire, a 25-item self-reported instrument developed by Meikle et al (2012), was used to determine the presence of tinnitus. This survey includes 8 subscales: Intrusive, sense of control, cognitive, sleep, auditory, relaxation, quality of life, and emotional to characterize the severity of tinnitus and assess its impact. Each subscale contains 3 questions, whereas quality of life subscale contains 4 questions. The total score ranges from 0 to 100. This survey has been demonstrated to have an excellent test-retest reliability of 0.78 and high internal consistency reliability with a Cronbach’s alpha value of 0.97 [10,11]. The TFI survey has been used previously to assess the severity of tinnitus in firefighters [6]. Therefore, we employed the same survey to enable the comparison of our findings with these studies. 

### 2.5. Data Analyses

Linear regression analyses were used to determine the relationships between age, number of years in service as a firefighter, self-perceived hearing handicap (HHIA score), and the severity of tinnitus (TFI score). A bivariate regression analysis was employed to determine the association of: (1) age with HHIA and TFI scores; (2) number of years in service with HHIA and TFI scores; and (3) HHIA with TFI scores, precluding the necessity to correct for multiple comparisons. The data was analyzed in Microsoft Excel using Data Analysis Toolkit (Office Professional Plus 2016, Microsoft, Redmond, WA, USA). A *p*-value of <0.05 was considered to indicate a statistically significant association between the variables. The results are expressed as mean ± standard deviation. 

## 3. Results

### 3.1. Characteristics of Study Participants

Demographic characteristics of firefighters in our study are shown in Table 1. Among the 42 firefighters who participated in this study, more than 95% were male. This is reflective of the gender distribution in this fire department, which has approximately 821 male and 12 female firefighters. The average age of the participants was 48 years and ranged between 35 and 59. Analysis of the race/ethnicity distribution indicated that 64% of the participants were Caucasians while 36% were African Americans. On average the participants had worked as firefighters for 23 years, with a range of 12 to 38 years. Approximately, 5% of the firefighters in the department participated in this study.

### 3.2. Hearing Handicap

**Figure 1:**
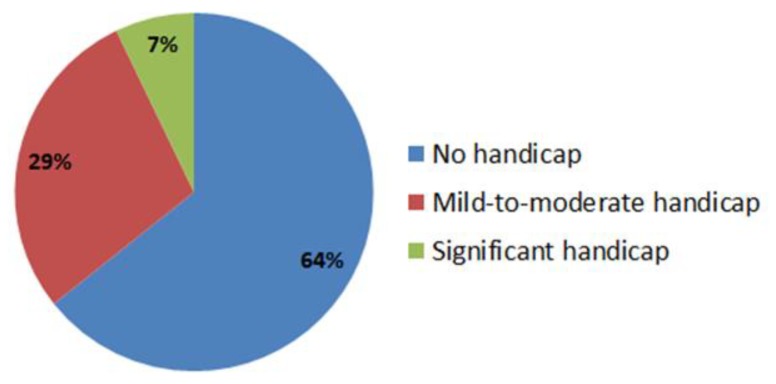
Self-perceived hearing handicap in firefighters. The hearing handicap inventory for adults (HHIA) scores for 27 firefighters were in the 0–17 range, suggesting no hearing handicap. However, the HHIA scores for 12 firefighters were in the 18–42 range, suggesting mild-to-moderate handicap and the HHIA scores for 3 firefighters were above, 42 suggesting significant hearing handicap.

Self-perceived hearing handicap, assessed using the HHIA scores, indicated that 64% of firefighters had no handicap, 29% had a moderate handicap, and 7% had a significant handicap (Figure 1). Scores in the range 0–17 was considered as “no hearing handicap,” 18–42 as “mild-to-moderate handicap,” and >43 as “significant handicap.” The mean score in this cohort was 13.24 (median = 7) and ranged from 0 to 56 (Table 2). Further analysis of the social and emotional subscales showed that the mean score was 12.50 (0–58.33) and 13.74 (0–57.69), respectively. Our results indicated that a third of participants had some degree of hearing handicap.

### 3.3. Tinnitus

**Figure 2:**
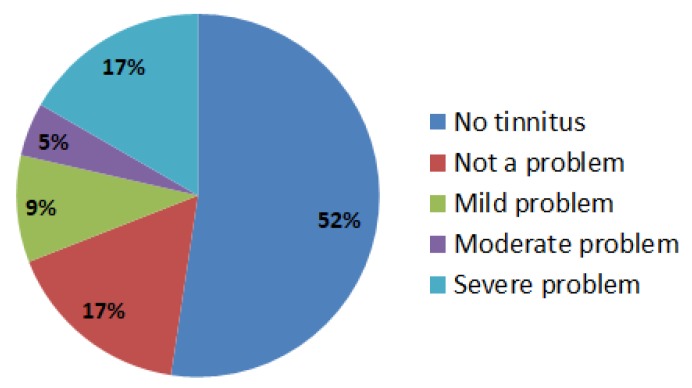
Tinnitus in firefighters. The tinnitus functional index (TFI) score of 22 firefighters was 0 (no tinnitus) and for 7 firefighters it was in the 0–9 range, suggesting that tinnitus was not a problem. The TFI score of 4 firefighters was in the 10–20 range and for 2 firefighters it was in the 21–29 range, suggesting that tinnitus was a mild-to-moderate problem. However, the TFI score of 7 firefighters was above 30 suggesting that tinnitus was a severe problem.

The presence of tinnitus was determined using the “History of Prior Exposure” survey, which specifically inquired whether the firefighter has a ringing noise in the ear (Question #7). The severity of tinnitus was assessed using the TFI questionnaire. Among the 42 firefighters who participated in this study 52% did not report tinnitus, 17% did not perceive tinnitus as a problem, 9% perceived tinnitus as a mild problem, 17% perceived tinnitus as a mild to moderate problem, and 5% perceived tinnitus as a moderate problem or greater (Figure 2). Scores in the range 0-9 were considered “not a problem,” 10–20 as a “very mild problem,” 21–29 as a “moderate problem,” and equal to 30 or more as a “severe problem.” The mean TFI score in this cohort was 11.47 (median = 0) and the range was from 0 to 81 (Table 3). Analysis of subcategory scores indicated that tinnitus had some interference with auditory functions in 15 firefighters, altered cognitive functions in 14, was intrusive and interfered with sleep and relaxation in 12, altered the sense of control in 11, affected the quality of life in 10 and the emotional state in 9 participants. 

### 3.4. Association between Hearing Handicap and Tinnitus

**Figure 3:**
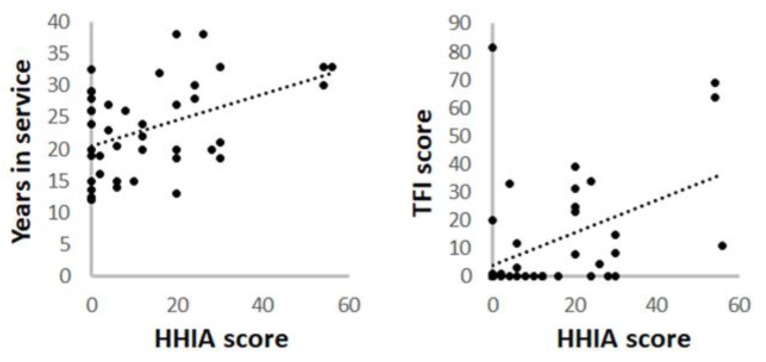
Association of self-perceived hearing handicap with duration of employment and tinnitus. Bivariate regression analysis indicated a positive correlation between HHIA scores and the number of years in service as a firefighter (R^2^ = 0.1926, left panel). The strength this correlation increased when controlled for age (R^2^ = 0.2225). In addition, the HHIA scores were associated with the TFI scores (R^2^ = 0.1975, right panel).

We examined the association between different variables by using linear regression analysis. Age and years in service were considered as independent variables while tinnitus and hearing handicap were considered as dependent variables. Age was not associated with the presence of tinnitus and hearing handicap (*p*-value = 0.25 and 0.07, respectively). Though the number of years in service was not associated with tinnitus (*p*-value = 0.14) it was associated with hearing handicap (*p*-value = 0.004; Figure 3). More importantly, hearing handicap was significantly associated with the presence of tinnitus in firefighters (*p*-value = 0.003; Figure 3). 

## 4. Discussion

Firefighting is a hazardous job with significantly high risk of work-related injuries. Good hearing is essential for firefighters to take appropriate and timely action in critical situations, and tinnitus has the potential to interfere with their life-saving actions. In this study a cohort of 42 firefighters from the Detroit metropolitan area was recruited to assess the incidence of tinnitus. Results show that a third of the firefighters have some degree of difficulty in hearing and that 31% have tinnitus with different degrees of severity. In addition, we found a statistically significant association between years in service and hearing handicap and between hearing handicap and tinnitus. Considering that tinnitus is found in 10–15% of adults [12], our results suggest that the prevalence of hearing handicap and tinnitus in firefighters is twice as that of the general population.

Subjective tinnitus is characterized by the perception of phantom sound, without external acoustic stimuli, that can be heard only by the afflicted person. It affects between 10% and 15% of the adult population all over the United States and Europe. It is stressful and very damaging to the overall health of patients. Those who have tinnitus complain of various symptoms commonly associated with the condition, such as sleep deprivation, difficulty in working, depression, anxiety, and various other health impairments [13,14]. A strong relationship between tinnitus severity and depression or anxiety was found in patients with chronic tinnitus [15]. In addition to affecting the quality of life, tinnitus is likely to affect the ability to concentrate, which is critical for firefighting.

Along with loud noise exposure, firefighters may be exposed to multiple ototraumatic pollutants every day. Heavy metal exposures, with or without noise overexposure, are most frequently associated with hearing loss and tinnitus [2,16]. A study of 2031 men and 311 women belonging to the Australian Defense Force Personnel indicated that daily exposure to more than one toxic agent, such as loud noises, heavy metals, intense smoke, engine exhaust, solvents, and chemical spills, increased the risk of moderate or severe tinnitus. The participants self-reported the type of damaging exposure by answering a questionnaire. Exposure to two types of pollutants increased the risk of moderate to severe tinnitus twofold. If four pollutants were involved the risk increased fourfold [17]. Because of the old housing stock and the auto industry in Detroit firefighters are probably exposed to higher levels of heavy metals such as lead and cadmium, which can cause auditory dysfunction [18,19]. The length of occupational exposure to environmental pollutants appears to be a predictor of hearing problems in firefighters, as indicated by the association of hearing handicap with the number of years in service. The hearing handicap among firefighters also depend on their specific role, because the exposure type and level varies accordingly. Hearing loss was found to be more pronounced in rescuers (25.8%) than in office workers, suppressors, paramedics, and drivers (16.6–13.8%) [20]. However, an association between the number of years in service and tinnitus was not detected, probably because of the smaller size of this study. Nevertheless, a significant association between self-perceived hearing handicap and tinnitus was detected in our study population. This finding is consistent with previous studies, which indicate that subjective tinnitus is often associated with hearing loss [21] and that firefighters who have some kind of hearing impairment more likely have tinnitus as well [22]. 

The strength of this study is that it focuses on a specific population that is continuously exposed to multiple ototraumatic agents that are commonly found in urban communities. However, there are some limitations that should be considered while interpreting the findings of this study. First, the number of participants was relatively low, as the total size of our target population is small. Second, because the participation in this study was voluntary, the sample may be biased. For example, firefighters with considerable hearing difficulties may not be inclined to reveal their health issues and may not have volunteered. Third, although African American firefighters represented about 36% of the participants, this study lacks representation from other minority groups, such as Hispanics, Asians, and Native Americans. Finally, the prior exposures survey is prone to recall bias and some data collected in this study may be subjective, as it is based on a self-assessment of difficulties in hearing and problems associated with tinnitus. Nevertheless, the findings of this study could be extrapolated to those who are exposed to multiple ototraumatic agents in other occupational settings and urban localities.

## 5. Conclusions

Firefighters are at risk of hearing loss and tinnitus, probably due to increased exposure to multiple ototraumatic agents in the firefighting environment. The significant associations between number of years in service and hearing handicap, and between hearing handicap and tinnitus, highlight the importance of preventive measures in limiting exposure to occupational risk factors. The findings of this study would be useful for directing efforts to advocate for better occupational health policy and minimize the incidence of hearing loss and tinnitus in firefighters.

## Figures and Tables

**Table 1:** Basic demographic characteristics.

Demographic Characteristics	Firefighters (*n* = 42)
Sex	
Male	40 (95.2%)
Female	2 (4.8%)
Race/ethnicity	
African American or Black	15 (33.7%)
Caucasian	27 (64.3%)
Age (year)	
Mean ± SD	47.95 ± 6.77
Median	48.5
Years as firefighter	
Mean ± SD	23.16 ± 7.30
Median	22.5

**Table 2:** Self-perceived hearing handicap scores.

Hearing Handicap Inventory for Adults (HHIA)	Firefighters (*n* = 42)
Mean ± SD	13.24 ± 15.55
Range	0–56
Subscales	
Social	
Mean ± SD	12.50 ± 15.37
Range	0–58.33
Emotional	
Mean ± SD	13.74 ± 16.97
Range	0–57.69

**Table 3:** Tinnitus functional index scores.

Tinnitus Functional Index (TFI)	Firefighters (*n* = 42)
Mean ± SD	11.47 ± 20.24
Range	0–81.6
Subscales	
Intrusive	
Mean ± SD	14.09 ± 24.86
Range	0–93.33
Sense of control	
Mean ± SD	11.94 ± 22.68
Range	0–76.67
Cognitive	
Mean ± SD	12.3 ± 25.27
Range	0–100
Sleep	
Mean ± SD	11.59 ± 23.40
Range	0–80
Auditory	
Mean ± SD	15.79 ± 27.21
Range	0–80
Relaxation	
Mean ± SD	13.17 ± 25.81
Range	0–100
Quality of life	
Mean ± SD	8.15 ± 18.46
Range	0–70
Emotional	
Mean ± SD	5.24 ± 13.19
Range	0–63.33
